# Seroprevalence and Modifiable Risk Factors for *Toxocara* spp. in Brazilian Schoolchildren

**DOI:** 10.1371/journal.pntd.0002830

**Published:** 2014-05-29

**Authors:** Alex J. F. Cassenote, Alba R. de Abreu Lima, José M. Pinto Neto, Guita Rubinsky-Elefant

**Affiliations:** 1 Postgraduate Program in Infectious and Parasitic Diseases, Faculty of Medicine, University of São Paulo, Cerqueira César, São Paulo, Brazil; 2 Faculty of Medicine of São José do Rio Preto, Department of Molecular Biology, Vila São José, São José do Rio Preto, Brazil; 3 Faculty of Health Sciences, University Camilo Castelo Branco, São Paulo, Brazil; 4 Institute of Tropical Medicine of São Paulo, University of São Paulo, Cerqueira César, São Paulo, Brazil; University of Saskatchewan, Canada

## Abstract

**Background:**

Toxocariasis is a worldwide helminthic zoonosis caused by infection with the larvae of the ascarid worms that comprise the *Toxocara* spp. Children are particularly prone to infection because they are exposed to the eggs in sandboxes and playgrounds contaminated with dog and cat feces. Certain behaviors, such as a geophagy habit, poor personal hygiene, a lack of parental supervision, close contact with young dogs, and ingestion of raw meat, as well as gender, age, and socioeconomic status, affect the prevalence of the disease. However, previous studies of the risk factors for toxocariasis have generally produced inconsistent results. An epidemiological cross-sectional study was conducted to evaluate the seroprevalence of IgG anti-*Toxocara* spp. antibodies and associated factors in schoolchildren from a region in the southeast of Brazil.

**Methodology/Principal Findings:**

A total of 252 schoolchildren aged 1 to 12 years (120 males and 132 females) were assessed. An enzyme-linked immunosorbent assay based on *Toxocara canis* larval excretory-secretory antigens was used to determine outcomes. A questionnaire was used to collect information on children, family, and home characteristics. Clinical and laboratory data completed the dataset investigated in this study. Seroprevalence was 15.5% (95%CI 11.5–19.8). Geophagy (aPR 2.38 [95%CI 1.36–4.18], *p-value* 0.029) and the habit of hand washing before meals (aPR 0.04 [95%CI 0.01–0.11], *p-value* ≤0.001) were factors associated with increased and decreased seroprevalence, respectively. The income factor and its related variables lost statistical significance after adjustment with a multiple Poisson regression model.

**Conclusions/Significance:**

The current study confirms that toxocariasis is a public health problem in the evaluated area; modifiable factors such as soil contact and personal hygiene appear to have a greater influence on the acquisition of infection than sociodemographic attributes, thus representing direct targets for disease prevention and control.

## Author Summary

Human toxocariasis is a neglected parasitic infection occurring in Brazil and worldwide. The combination of the close proximity of canines and felines to humans, environmental contamination by infectious forms of the parasite and neglect by public health officials provides a favorable situation for the spread of this zoonosis, which primarily affects pediatric populations. An epidemiological study in an area of known parasitic soil contamination was conducted to evaluate the impact of this disease and the factors that influence its occurrence. A sample of 1- to 12-year-old children was obtained with random selection of schools, classrooms and schoolchildren. A questionnaire was used to collect socioeconomic and behavioral information, laboratory tests determined the presence of antibodies against *Toxocara* spp. parasites and a medical evaluation noted clinical features of the disease. The study found that antibodies is present in more than 15% of subjects and can be in as many as 30% depending on individual characteristics. The analysis also indicated that behaviors that increase soil ingestion, such as nail biting, eating dirt or poor personal hygiene, contribute to increased frequency of the disease and may be even more important than the socioeconomic status of individuals. These behaviors therefore represent real and simple targets for the control of this neglected tropical disease.

## Introduction

Human toxocariasis is a helminthic zoonosis caused by the larvae of the ascarid worms of *Toxocara* spp. In the early 1950s, *Toxocara canis* was recognized as a human pathogen [1], and the term “visceral larva migrans” was so widely used that human toxocariasis is also known as visceral larva migrans syndrome. Two species of roundworms, *Toxocara canis* and *Toxocara cati,* are recognized as causative agents of human toxocariasis [2],[3],[4],[5]. The adults of both of these species parasitize the small intestines of their definitive hosts, which are canids and felids, respectively [6].

*Toxocara* spp. have a worldwide distribution and tend to be more prevalent in tropical regions, including industrialized countries, where they are considered the cause of the most frequent form of helminthiasis. Toxocariasis has been described as the most common human parasitic worm infection in developed countries [2],[7],[8].

Humans are infected via the accidental ingestion of embryonated eggs containing *Toxocara* spp. larvae. This ingestion most commonly results from contact with contaminated soil and rarely from the ingestion of undercooked meat containing *Toxocara* larvae. Children are notably susceptible to infection because they are exposed to the eggs during recreational activities in sandboxes and playgrounds contaminated with dog and cat feces [2],[8],[9].

The clinical manifestations of toxocariasis are related to the location and degree of damage to host tissues caused by the inflammatory response to larval migration. These manifestations can vary from asymptomatic infection to the most severe: visceral toxocariasis (VT), characterized by larval migration through major organs including the liver, lungs and, more rarely, the central nervous system (CNS) [8],[10],[11]; ocular toxocariasis (OT), which occurs when the larvae of the parasite affect the human eye, causing severe inflammation and potentially producing partial or total loss of vision [12]; and less severe syndromes, characterized by nonspecific signs and symptoms, which have been primarily described in children. These syndromes include covert toxocariasis [13] and common toxocariasis [14] in adults.

The literature reports that behaviors such as geophagy, poor personal hygiene and a lack of parental supervision, close contact with young dogs and ingestion of raw meat as well as gender, age, and socioeconomic status affect the frequency of the disease [7]. Nevertheless, the results of various studies of the risk factors for toxocariasis have generally been inconsistent [15],[16].

Between 2007 and 2008, soil contamination by eggs of soil-transmitted helminths was evaluated in the same area investigated in the current study [17]. The authors observed a high rate of soil contamination by parasites with zoonotic potential (30.2%), including *Toxocara* spp. (79.3%), *Trichuris* spp. (13.8%) and *Ancylostoma* spp. (6.9%) in the evaluated samples from sandboxes and playgrounds located in public squares, an observation that prompted a serological evaluation of children in the region. The aim of this paper is to present the methodology and preliminary results of a cross-sectional study conducted to estimate the seroprevalence of IgG anti-*Toxocara* spp. and associated factors in schoolchildren aged 1 to 12 years from a region in southeast Brazil.

## Methods

### Scenario, study and sample design

**Figure 1:**
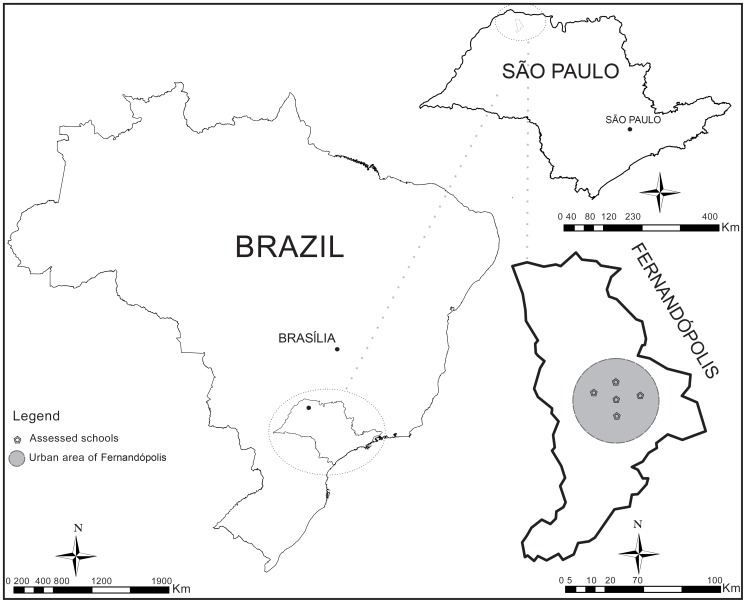
Geographic location of the study area. São Paulo is located in southeast Brazil, and Fernandópolis is situated in the northwest of São Paulo State.

Between 2007 and 2010, a cross-sectional study was conducted in urban areas of Fernandópolis (S.20°17'02'' W.50°14'47''), a city with 59,580 inhabitants located in the northwestern São Paulo State in the southeast of Brazil (Figure 1). Fernandópolis has a high Human Development Index (a comprehensive statistic that incorporates population life expectancy, education and income) of 0.83, whereas the HDI value for Brazil is 0.71 [18].

The population of individuals aged 1 to 12 years in the city was estimated to total approximately 22,000 children studying in 31 schools, 18 of which were public (funded by the state or municipal government, with no cost to students), 8 philanthropic (funded in whole or in part by churches, with no cost to students), and 5 private schools (self-funded by charging their student body) [19].

The sample size (n = 252) was based on the following parameters: 95% confidence level and a 5% margin of error, a statistical power of 80%, design effect of 1.5, and an estimated prevalence of 12.4% (average proportion of two sampling strata), as observed by Campos Junior et al. [20].

A complex sample (multi-stage cluster and strata sampling) [21],[22] was prepared. In the first stage, the number of schools with students within the age range being studied was identified (n = 31 schools), and then, schools were selected with a probability proportional to the type of school (public, philanthropic, or private). In total, 2 public schools, 2 philanthropic schools and 1 private school were selected. In the second stage, simple random sampling was used to select groups, the number of classrooms with students within the age range under study was identified (n  =  40 classrooms), and 25 classrooms were selected (5 classrooms for each school) with a probability proportional to the eligible age ranges. Three operational strata were used: 1 to 4, 5 to 8, and 9 to 12 years old. The representativeness of the age groups was thus conserved intra-classroom. The children outside the specific age group within a classroom were excluded from the final stage, in which children were selected within each classroom by simple random sampling with replacement (n = 10 children per classroom for original sample members and n = 3 children per classroom for replacement sample members). Additionally, 2 children were randomly selected from the 25 classrooms to fulfill the prespecified sample size of 252. In this stage, the sampling also preserved 2 income strata (with similar population proportions): less than or equal to 2 minimum wages were included in stratum stA, and incomes higher than 2 minimum wages were included in stratum stB.

The replacement sample members were identified as alternates and placed on a waiting list; they were included in the study if original sample members refused to participate. The process of identifying the classrooms and the students was based on an updated list of the students in each participating school, with birth dates previously provided by the 5 schools that participated in the study. This list permitted all procedures to be performed randomly.

### Data collection, variables, outcome definitions and ethical considerations

After receiving a formal request and information on the importance, objectives and methodology of this study, the school board of each selected school gave permission for the study to be conducted. Selected children and their families were approached by interviewers to validate the prospective subjects' consent to participate in this research, and over the next week, the interviewers visited the families to collect data and schedule a medical visit for the child at the Support Center for Infectious and Parasitic Diseases in Fernandópolis.

Some of the variables investigated in blocks were as follows: *1) characteristics of children (collected from family interview questionnaire):* gender, age, type of school (public, philanthropic, or private), geophagy, onychophagy and hand-washing habits; *2) family and home characteristics (collected from family interview questionnaire):* SUS users only (SUS - Brazilian Unified Health System) and income (wage of head of household); *3) clinical characteristics (collected from medical records):* history or presence of hepatomegaly, splenomegaly, adenomegaly, cutaneous manifestations (urticarial, pruritus, or eczema), lung manifestations (wheezing, bronchitis, or cough), pneumonia, and seizures; *4) laboratory characteristics (collected from laboratory reports):* complete blood count (CBC; absolute and relative eosinophils, and other cells), intestinal parasites (larvae and eggs in fecal samples), and anti-*Toxocara* spp. IgG antibodies evaluated with an enzyme-linked immunosorbent assay (ELISA).

Data from different sources were collected by a team of 8 interviewers who were previously trained in pilot studies over a 1-week interval at 2 schools that were not included in the final sample. The kappa coefficient was used to verify agreement between the 2 questionnaire applications. The agreement between the questionnaire applications was considered very good (kappa coefficient = 0.94). Refusal at the blood collection or interview stage was observed in 3.17% (8) of individuals without prejudice to the study because the alternate participants were then included in the study.

For this specific study, 2 binary (yes/no) outcomes were considered: (1) *positivity by ELISA test* for anti-*Toxocara* spp. antibodies, described in the following methodology sections, and (2) *physician diagnosis* that was performed by an infectious and parasitic diseases expert based on the clinical history of characteristic signs and symptoms (described in item 3 of this section) and the assessment of laboratory findings, the most relevant of which are the serology (ELISA anti-*Toxocara* spp.) and eosinophilia (from CBC). As the main objective of the study was to evaluate the risk factors for contact with the parasite, greater emphasis was placed on the factors associated with the first outcome.

The study was reviewed and approved by the Research Ethics Committee of the Clinics Hospital, São Paulo University Medical School (Protocol Number #0518-07), in accordance with Brazilian and international laws. Legal guardians were informed about the study ethical criteria and procedures by letter and by interview with the research assistant; they all signed the informed consent for the children that participated in this study.

### Blood collection, complete blood count (CBC) and parasitological examination of stools

Blood samples were collected by the Clinical Laboratory School from the Fernandópolis Educational Foundation into Vacutainer™ tubes (BD, Franklin Lakes, NJ, USA) with and without EDTA. The blood in the EDTA tubes was used for CBC counts and differentiation. Red and white cell counts were obtained with a Coulter T890 automated hematology analyzer (Beckman Coulter, Brea, CA, USA), and differential leucocyte counts were evaluated via the microscopic examination of stained blood smears. Eosinophil levels (to characterize relative eosinophilia) were assessed as the percentage of total leucocytes represented by eosinophils [23].

Serum samples were separated from the blood in EDTA-free tubes by centrifugation and stored at −20°C prior to use. The samples were sent to the Seroepidemiology and Immunobiology Laboratory of the São Paulo Tropical Medicine Institute for the IgG anti-*Toxocara* spp. ELISA test.

A series of stool samples for parasitological examinations were collected from the schoolchildren on 3 different days in the same container with the liquid preservative MIF (merthiolate-iodine-formaldehyde). The following parasitological methods to detect the larvae and eggs of parasites were used in the laboratory analyses: the flotation technique in a saturated sodium chloride solution with a density of 1.20 g/mL [24], the spontaneous fecal sedimentation technique [25], and the formol-ethyl acetate concentration technique [26].

### Antigen preparation, serum processing and antibody detection

A preparation of infective *Toxocara canis* larval excretory-secretory antigens (TES) was obtained as described by Rubinsk-Elefant et al. [27], with several modifications briefly described as follows. Eggs obtained from the uteri of female worms were incubated in 2% formalin for approximately 1 month at 28°C. Formalin was removed after exhaustive washing with physiological sodium chloride solution (0.85% NaCl), and the embryonated eggs were artificially hatched in serum-free Eagle's medium. Larvae were recovered by transferring the suspension on a loose cotton wool plug to a Baermann apparatus. After 18 h, larvae were collected from the bottom of the apparatus, and cultures were incubated at 37°C. The supernatants that contained the antigens were removed and replaced with fresh Eagle's medium at weekly intervals. Different batches were pooled, concentrated in an Amicon apparatus, dialyzed against distilled water, centrifuged (18,500 g) at 4°C for 60 min, and filtered with a 0.22 mM Millipore membrane (Millipore, Danvers, MA, USA). The protein content was determined with the Lowry protein assay [28]. Antibodies cross-reacting to *Ascaris* spp. were removed from the sera by preincubating with an adult worm extract (AWE) of *Ascaris suum* [27] [29]. In the enzyme-linked immunosorbent assay (ELISA), all sera were preincubated with a solution (25 µg/mL) of AWE in 0.01 M phosphate buffered saline (PBS, pH 7.2) containing 0.05% Tween-20 (PBS-T) for 30 min at 37°C. Ninety-six-well microtitration polystyrene plates (Corning, Costar, New York, NY) were coated (100 µL/well) with TES antigen solution (1.9 mg/mL in a 0.1 M carbonate–bicarbonate buffer, pH 9.6), incubated for 2 h at 37°C, and then incubated for 18 h at 4°C in a humidified chamber. After the wells were washed 3 times with 0.01 M (PBS, pH 7.2) containing 0.05% Tween-20 (PBS-T), the plates were blocked (200 µL/well) with 1% bovine-serum albumin in PBS-T (BSA, Sigma, St. Louis, MO, USA) for 1 h at 37°C. After 3 washing cycles with PBS-T, the plates were incubated (100 µL/well) with serum samples (dilution: 1/320) for 40 min at 37°C. After incubation with serum samples, the plates were washed 3 times and incubated (100 µL/well) with horseradish peroxidase-conjugated goat anti-human IgG (Sigma) at a 1∶10,000 dilution in PBS-T for 40 min at 37°C. The plates were washed 3 times and incubated (100 µL/well) with ortho-phenylenediamine (0.4 mg/mL, OPD-Fast, Sigma, Dorset, United Kingdom) and H_2_O_2_-urea (0.4 mg/mL) in 0.05 M citrate buffer for 15 min at 37°C. The reaction was stopped (50 µL/well) with 2 M H_2_SO_4_. The assay was monitored by including negative and positive serum samples in addition to a blank (no serum sample). Absorbance at 492 nm was determined in an automatic microplate reader (Titertek Multiskan MCC/340, Lab-system, Helsinki, Finland). A cut-off absorbance value was defined as the mean absorbance reading for 96 negative control sera plus 3 standard deviations. Antibody levels were expressed as reactivity indices (RIs), which were calculated as the ratio between the absorbance values of each test sample and the cut-off value; positive samples had RIs greater than 1.

### Database and statistical analysis

Data were entered by 2 research assistants on a specific EpiData 3.1 form (The EpiData Association, Odense, Denmark) using the double entry method with subsequent validation. The agreement observed was high (kappa = 0.92), and inconsistencies were resolved to complete agreement (kappa = 1.00). The database was exported to the Statistical Package for the Social Sciences (SPSS) 20 for Windows (International Business Machines Corp, New York, USA), to STATA 12 (StataCorp LP, Texas, USA), and R version 3.0.2 (http://www.r-project.org/) for statistical treatments.

The selected variables were initially studied in conjunction with confidence intervals (CI) estimated from 1,000 bootstrap samples [30]. The design effect (*deff*) was estimated to study the variance between and within the clusters (classrooms) in relation to the income sampling stratum for all the presented variables [31],[32],[33],[34]. A post-hoc proportion Z-test was conducted between sample and population categories [35]. The variables analyzed were gender, age, income (wage of head of household), and school type.

A univariate analysis was performed with the Pearson chi-squared test (χ2), while a chi-square test for linear trends or Fisher's exact test were initially used to examine the association between positivity in the IgG anti-*Toxocara* spp. test and the analyzed factors [36]. A crude prevalence ratio (*c*PR) was applied to assess the impact of individual factors on outcomes [37]. A multiple Poisson regression with robust variance was used to estimate the adjusted prevalence ratio (*a*PR), and a 95% confidence interval (95% CI) was also applied [38],[39],[40], as recommended for high-prevalence outcomes [41],[42] in conjunction with consideration of *deff* [43],[44]. The variables significantly associated in the univariate model (p-*value* <0.05) remained in the final models (all entered simultaneously). To improve the final model, the predictor variables were tested for collinearity with variance inflation factor (VIF) and for the presence of influential values. The accuracy of the model was evaluated using a cross-validation system [45],[46]. The significance level was set at p-*value* <0.05.

## Results

The general prevalence of IgG anti-*Toxocara* spp. antibodies was 15.5%, with equal variability between and within clusters considering income strata (*deff* 1.0). The descriptive analyses of the evaluated attributes, socio-demographic characteristics (including sample and population comparisons), behaviors that increase soil ingestion, and clinical laboratory characteristics including the prevalence of IgG anti-*Toxocara* spp. antibodies are shown in Table 1.

**Table 1:** Proportions with a 95% confidence interval (95% CI) pertaining to the characteristics of 252 schoolchildren aged 1 to 12 years from urban areas of Fernandópolis, in the northwest of São Paulo State, Brazil.

	Absolute frequency	% (95% IC †)	DEFF ˇ	Population (%)‡	p-*value* ‡‡
***Socio-demographic characteristics***					
Gender					
Female	132	52.4 (46–57.9)	1.0	50.7	0.821
Male	120	47.6 (42.1–54)	1.0	49.3	0.179
≥9					
6 |- 9	47	18.7 (13.9–23.0)	1.6	21.9	0.771
4 |- 6	69	27.4 (22.2–32.5)	1.9	25.9	0.178
< 4	88	34.9 (29.4–41.3)	1.5	31.3	0.223
Overall	48	19.0 (13.9–23.8)	1.7	20.9	0.867
Income (head of household)					
≥5	67	26.6 (21.4–32.1)	1.6	25.4	0.708
4 |- 5	37	14.7 (10.3–19.4)	1.7	9.9	0.802
3 |- 4	15	6.0 (3.6–9.1)	1.5	7.4	0.810
2 |- 3	13	5.2 (2.4–7.9)	1.1	13.9	0.445
1 |- 2	70	27.8 (22.6–33.3)	1.1	20.2	0.103
<1	50	19.8 (15.1–25.0)	0.8	23.2	0.353
Type of School					
Private	103	40.9 (34.9–46.8)	1.9	35.5	0.094
Public and philanthropic	149	59.1 (53.2–65.1)	1.9	64.5	0.081
SUS users only††					
Yes	99	39.3 (32.9–45.6)	1.8	-	-
No	153	60.7 (54.4–67.1)	1.8	-	-
***Behaviors that increase soil ingestion***					
Geophagy £					
Yes	85	33.7 (28.2–40.1)	0.9	-	-
No	167	66.3 (59.9–71.8)	0.9	-	-
Onychophagy £					
Yes	96	38.1 (32.1–43.7)	1.8	-	-
No	156	61.9 (56.3–67.9)	1.8	-	-
Hand washing habitµ					
Yes	193	76.6 (71.4–81.3)	0.9	-	-
No	59	23.4 (18.7–28.6)	0.9	-	-
***Clinical laboratory characteristics***					
ELISA anti-*Toxocara* spp. antibodies					
Positive	39	15.5 (11.5–19.8)	1.0	-	-
Negative	213	84.5 (80.2–88.5)	1.0	-	-
Relative eosinophilia					
Yes	53	21.0 (15.9–26.2)	1.4	-	-
No	199	79.0 (73.8–84.1)	1.4	-	-
Physician diagnosis ¥					
Yes	20	7.9 (4.8–11.5)	1.6	-	-
No	232	92.1 (88.5–95.2)	1.6	-	-

ˇ Design effect considering income strata.

† The % refers to the distribution in the studied sample. The confidence interval (95% CI) was estimated based on 1,000 bootstrap samples.

†† Refers to the families who have no health insurance at their disposal and are solely and exclusively dependent on the Brazilian Unified Health System.

£ Note these habits at least 1 time in the past month.

µ Note the habit of hand washing before meals (at least 3 times a day).

¥ Based on clinical presentation (signs and symptoms) and laboratory results (serology and eosinophilia).

‡ Relative frequency in the Fernandópolis population.

‡‡ Proportion Z-test comparing sample vs. population.

-There are no population data available.

The variable gender showed an equal distribution in the sample, already age showed high percentages for the categories 4 |- 6 years and 6 |- 9 years. Income was concentrated in the first category, <1 minimum wage, and in the last category, ≥5 minimum wages. The public and philanthropic categories for the type of school were combined due to the similarity of individuals and the representativeness of the sample. The category of exclusive users of SUS completed the socio-demographic characteristics block. For the socio-demographic variables (excluding SUS users only), all the tested attributes were found to be comparable to the statistical distribution in the population. The data on behaviors that increase soil ingestion showed that participants without geophagy, without onychophagy, and with the habit of hand washing dominated the sample (Table 1).

Prevalence according to the sampling strata was studied for two possible outcomes, IgG anti-*Toxocara* spp. antibodies and physician diagnosis, with statistical significance (*p-value* ≤ 0.001) observed for both variables and a greater impact on stA in both cases (Table 2).

**Table 2:** Crude prevalence ratio with a 95% confidence interval (95% CI) based on the sampling strata of schoolchildren aged 1 to 12 years from urban areas of Fernandópolis, in the northwest of São Paulo State, Brazil.

	Positive	%	cPR (95% CI)	p-*value*
ELISA anti-*Toxocara* spp. antibodies				
stA	34	28.3	7.48 (3.24–18.50)	
stB	5	3.8	1.00	<0.001†
Overall	39	15.5		
Physician diagnosis ¥				
stA	17	14.2	6.23 (1.87–20.74)	
stB	3	2.3	1.00	0.001††
Overall	20	7.9		

st – stratum.

cPR - crude prevalence ratio

† Chi-squared test

†† Fisher exact test

¥ Based on clinical presentation (signs and symptoms) and laboratory results (serology and eosinophilia).

The unadjusted analysis showed that factors associated with IgG anti-*Toxocara* spp. antibodies that contributed to higher prevalence were SUS users only, geophagy, and onychophagy. Relative eosinophilia was also suggested as an associated factor. Age was associated only considering a linear trend effect but was not significant as an estimator of risk. Income in the categories of ≥5 minimum wages, 4 |- 5 minimum wages, private school, and the habit of hand washing were factors that contributed to low prevalence for this outcome. After adjustment in the Poisson model, however, most analyzed variables lost their statistical significance and remained associated only with the habit of hand washing and geophagy (Table 3).

**Table 3:** Crude and adjusted prevalence ratio with a 95% confidence interval (95% CI) based on independent variables for schoolchildren aged 1 to 12 years from urban areas of Fernandópolis, in the northwest of São Paulo State, Brazil.

	Positive†	%	cPR (95% CI)	p-*value* ‡	aPR (95% CI)	p-*value* ‡‡
***Socio-demographic characteristics***						
Gender						
Female	17	14.2	0.85 (0.48–1.52)	0.584	1.27 (0.75–2.13)	0.357
Male	22	16.7	1.00		1.00	
Overall	39	30.8	-		-	
Age (years)						
≥9	5	10.6	0.43 (0.15–1.21)		0.66 (0.40–1.07)	0.094
6 |- 9	8	11.6	0.46 (0.19–1.14)	0.040	0.82 (0.43–1.50)	0.555
4 |- 6	14	15.9	0.64 (0.29–1.38)		1.27 (0.87–1.86)	0.195
<4	12	25.0	1.00		1.00	
Overall	39	63.1	-		-	
Income (head of household)						
≥5	1	1.5	0.05 (0.01–0.35)		0.21 (0.00–10.68)	0.438
4 |- 5	1	2.7	0.08 (0.01–0.64)		1.11 (0.02–54.50)	0.957
3 |- 4	2	13.3	0.42 (0.10–1.81)	≤0.001€	1.07 (0.21–5.42)	0.931
2 |- 3	1	7.6	0.24 (0.03–1.81)		0.53 (0.07–4.21)	0.548
1 |- 2	18	25.7	0.80 (0.41–1.58)		1.00 (0.45–2.23)	0.954
<1	16	32.0	1.00		1.00	
Overall	39	82.9	-		-	
Type of school						
Private	2	1.9	0.06 (0.1–0.22)	≤0.001	1.35 (0.04–52.71)	0.940
Public and philanthropic	37	24.8	1.00		1.00	
Overall	39	26.8	-		-	
SUS users only††						
Yes	34	22.2	4.40 (1.78–10.8)	≤0.001	0.82 (0.39–1.66)	0.560
No	5	5.1	1.00		1.00	
Overall	39	27.2	-		-	
***Behaviors that increase soil ingestion***						
Geophagy£						
Yes	28	32.9	5.00 (2.49–10.05)	≤0.001	2.38 (1.36–4.18)	0.003
No	11	6.5	1.00		1.00	
Overall	39	39.5	-		-	
Onychophagy£						
Yes	23	23.9	2.34 (1.23–4.42)	0.009	1.06 (0.63–1.78)	0.865
No	16	10.2	1.00		1.00	
Overall	39	34.2	-		-	
Hand washing habitµ						
Yes	3	1.5	0.03 (0.01–0.08)	≤0.001	0.04 (0.01–0.11)	≤0.001
No	36	61.0	1.00		1.00	
Overall	39	62.5	-		-	
***Clinical laboratory characteristics***						
Relative eosinophilia						
Yes	16	30.1	2.61 (1.38–4.94)	0.003	1.51 (0.78–2.91)	0.204
No	23	11.5	1.00		1.00	
Overall	39	41.7	-		-	

cPR - crude prevalence ratio (considering the design effect).

aPR - adjusted prevalence ratio (considering the design effect).

† ELISA anti-*Toxocara* spp. antibodies: positivity is the outcome.

†† Refers to the families who have no health insurance at their disposal and are solely and exclusively dependent on the Brazilian Unified Health System.

£ Note these habits at least 3 times in the past month.

µ Note the habit of hand washing before meals (at least 3 times a day).

‡ Pearson chi-squared test.

‡‡ Wald chi-squared test.

€ Fisher exact test.

Intestinal parasite infections or commensal species were detected in 33 (14.9%) children with prevalences of 9.7% for protozoa and 5.8% for helminths. The predominant species were *Entamoeba coli* (4.5%), *Giardia* spp. (4.1%) and *Strongyloides stercoralis* (3.1%). No significant association was observed between intestinal parasites and anti-*Toxocara* spp. antibody positivity (Table 4).

**Table 4:** Frequency of parasites and commensals found in parasitological stool examinations from schoolchildren aged 1 to 12 years from urban areas of Fernandópolis, in the northwest of São Paulo State, Brazil.

	Frequency†	% (CI95%)††	Anti-*Toxocara* spp. antibodies		p-*value* †††
			Positive	% (CI95%)††	
*Entamoeba histolytica/díspar*	1	0.4 (0.0 – 2.2)	0	0.0	-
*Entamoeba coli*	10	4.5 (2.3–7.9)	2	20.0 (3.5–51.9)	0.535
*Giardia spp.*	9	4.1 (2.0–7.3)	2	22.0 (3.9–56.2)	0.477
*Enterobius vermicularis*	1	0.4 (0.0 – 2.2)	0	0.0	-
*Ascaris lumbricoides*	2	0.9 (0.1–2.9)	0	0.0	-
*Strongyloides stercoralis*	6	3.1 (1.1–5.5)	1	16.0 (0.8–59.1)	0.662
*Ancylostoma spp.*	4	1.8 (0.5–4.3)	1	25.0 (1.2–75.7)	0.603
Total	33	14.9 (10.6–20.1)	6	18.2 (7.7–34.3)	0.519

† Based on 221 analyzed samples.

†† Based on Exact Mid-P.

††† Based on Fisher exact test.

## Discussion

The seroprevalence of *Toxocara* spp. in schoolchildren aged 1 to 12 years from urban areas of Fernandópolis, in northwestern São Paulo State, was examined with an anti-*Toxocara* spp. ELISA test and estimated as 15.5% (95% CI 11.5–19.8). In previous studies of human toxocariasis in Brazilian children based on use of the same method, higher rates were reported: 51.6% [47] and 36.8% [48]. Other studies, such as Manini et al. [49], demonstrated rates (17.8%) similar to that found in the current study. Worldwide, *Toxocara* seroprevalence (based on an ELISA test) in children has been reported to range from 7.3% to 62.3% [50],[51],[52],[53],[54].

The sample investigated in this study showed good inferential quality for several reasons. First, the application of the resampling method helped to obtain narrower CIs to compare the sample with the population, and all CIs included the corresponding population parameter. Furthermore, no statistical significance was observed in the classic Z-test for proportions, also applied for this purpose. Thus, the sample showed similarities to several characteristics of the original population (compare the CI sample with the population proportion and the *p-values* in Table 1).

The impact of the complex sample design upon variance estimates is measured by the *deff*. It is defined as the ratio of the variance of a statistic that accounts for the complex sample design to the variance of the same statistic based on a hypothetical simple random sample of the same size.

Accordingly, a *deff* value of 1 indicates that the variance for the estimate under cluster sampling is the same as the variance under simple random sampling. In contrast, a *deff* value greater than 1 indicates that the effective sample size is less than the number of sampled persons but greater than the number of clusters; moreover, there is a loss of precision and a reduction in the effective sample size because individuals are chosen within clusters rather than sampled randomly throughout the population [22],[34]. In this study, a *deff* of 1.5 was used in the sample size calculation step to prevent a loss of precision, but an initial analysis demonstrated that the variables of age, income, type of school, SUS users only, onychophagy and physician diagnosis were associated with higher *deff* values. For this reason, the univariate and multivariate analyses incorporated the design effect to ensure more accurate inferences [43],[44].

In addition to the enabling environment for toxocariasis transmission observed in a previous study [17], the municipality under study has a large dog population. The most recent official data, based on a rabies vaccination campaign, reported approximately 18,000 dogs (an average based on the years 2008–2010) [55], corresponding to a ratio greater than 3 people for every dog. This phenomenon is difficult to measure and may contribute to the epidemiological context for toxocariasis in Fernandópolis.

In general, variation in the rate of seropositivity can occur due to the presence of dog and cat populations with high prevalences of *T. canis* and *T. cati* [56], close relationships between pets and humans indoors [57],[58],[59], and the defecation habits of pets or infected stray animals in streets and public squares. These factors produce contamination of the environment, particularly the soil, and can create an environment suitable for human infection [55],[56]. However, certain factors do not represent the life cycle of the parasite or the natural history of the disease but rather the research design and the outcome of the diagnostic method. First, the sampling design should consider the principles of randomness, uniformity and stratification of individuals relative to the group composition to ensure that the exposure factors and outcome found for the sample will be similar to the population. Considerable variation among laboratory methods, technical modifications in the production of TES, and disagreement over the cutoff definition must also be considered [27]. These factors can generate results that do not necessarily reflect the prevalence in the general population.

Differences in seropositivity for IgG anti-*Toxocara* spp. antibodies according to income strata have been found in previous studies. These differences always indicate a greater impact on the lower income classes [16],[20],[60]. The same effect was observed, although to a lesser extent, for the appearance of clinical disease (only VT cases were observed), but the reason for this outcome is that the basis of the medical diagnosis was the serologic test [3],[7],[8].

Other authors have stated that the duration of human IgG responses elicited by *Toxocara* larvae remains undetermined [61]. Viable larvae may persist in tissues and excrete/secrete antigens for several years, and no simple method is available to confirm parasite death after chemotherapy. Consequently, a single-sample IgG-ELISA titer cannot distinguish between past and current infection [62]. Based on this, the physician diagnosis was included in this evaluation to obtain more specific diagnoses of clinical disease. Thus, patients with a positive physician diagnosis received antiparasitic and/or anti-inflammatory therapy when needed, while those with positive serology without any apparent signs and symptoms were placed for clinical follow-up.

Many factors that predict *Toxocara* contact have been identified in human pediatric populations, but the results have been inconsistent [15],[29]. The results for gender have been contradictory. Specifically, the male gender has been observed to face increased risk [51],[57], to protect the subject [63],[64] and to show no association with *Toxocara* contact [52],[65],[66]. A young age [63], low socioeconomic status [16],[20],[60], low parental education [52], poor sanitation [53],[67], onychophagy [65],[66], geophagy [57],[59], an absence of hand washing before meals [54], eosinophilia [51],[58], and dog ownership [57],[58],[59],[68] are factors associated with *Toxocara* contact or infection. None of these known factors, except for geophagy and hand washing before meals, was significantly associated with *Toxocara* exposure in the population investigated in the current study, with *p-values* ranging between 0.094 and 0.954 in the final Poisson model (Table 3).

The significant associations between positivity of IgG anti-*Toxocara* spp. antibodies found in the univariate analysis, as well as the significant associations between this outcome and the magnitude of income, type of school, SUS users only, onychophagy, and relative eosinophilia, were lost after a multivariate analysis. The 2 variables that remained associated with this positivity variable represent the modifiable behavioral habits that underscore the proximity of humans to the parasite life cycle. The permanence of these in the multiple model, combined with the partial modification of geophagy and no modification of the habit of hand washing before meals (compare *p-values* and cPR vs. aPR in Table 3), suggests that income and the other sociodemographic attributes may be confounding factors in this relationship. The gender variable was retained in the final model, although it was not significantly associated in the univariate analysis because it implied a contradiction.

In practice, geophagy and a lack of hand washing before meals are behaviors that increase soil ingestion (directly or indirectly), facilitating human contact with the eggs of the parasites. We also suggest the hypothesis that hand washing before meals is more common in children whose socioeconomic status is high. For this reason, an association (due to a confounding effect) can be expected between poverty and the presence of IgG anti-*Toxocara* spp. antibodies. In summary, a confounding effect occurs if the association between exposure and outcome is distorted by the presence of another variable, the confounding factor [69],[70],[71]. Geophagy should be a phenomenon observed in both economic strata, in which hand washing demonstrates only a partial effect.

In general, income and related sociodemographic variables have been strongly emphasized as causal factors in the human-*Toxocara* relationship based on interpretations of unadjusted [20],[60],[72] or adjusted models, considering few [65],[73] or no [16],[74],[75] attributes that are important and directly related to the parasite life cycle, such as soil contact and personal hygiene. This paper, however, suggests that these modifiable factors are more important than sociodemographic attributes and are thus a direct target for disease prevention and control.

As a simple example, we suggest that modifiable factors discussed here, such as the habit of washing hands, can be taught within schools. A study in Brazil with students from elementary school addressed health education in toxocariasis prevention; among the most interesting findings, the researchers suggested that the use of only 1 approach is insufficient to change risk behaviors in children, which reinforces the idea that the educational process should be continuous throughout the stages of child development [76].

The strengths of this study are as follows: a complex probability sample with a multi-stage cluster design and stratified sampling of income, samples collected from different schools with the same methodology, and appropriate statistical analyses controlling for potential confounding factors and also consideration of the sampling design. The main limitations of this study is its cross-sectional design, which makes it impossible to establish causality and makes it difficult to relate the seropositivity found in the study to environmental contamination present in Fernandópolis.

In terms of the impact of sensitivity and specificity, we do not expect variation beyond that expressed by the confidence interval for the seroprevalence, especially because false positive results may occur due to trichinosis and fascioliasis, which are unusual infections in this population. Strongyloidiasis and other parasite infections was observed but were not significantly associated with serological status. The literature also reports that false negative results are rare and only occur in certain localized early or very old infections (OLM) [77]. A previous study found that the ELISA technique, accompanied by absorption of the serum with *Ascaris suum* antigens, demonstrated a sensitivity of 80% and a specificity of 90% [78].

For physician diagnosis, one laboratory parameter used by the physician was eosinophilia as determined from the CBC, which represented a non-specific test that could be altered for allergic conditions and parasitic infections. In this study, eosinophilia was used in conjunction with the presence of signs and symptoms and serology results to obtain a more specific clinical diagnosis.

Additional studies that consider a longitudinal perspective, such as a prospective-cohort design or statistical models employing hierarchical or multilevel analyses, are needed to make new contributions to the discussion of the factors related to contact with *Toxocara* spp., as the vast majority of previous research has involved cross-sectional studies using classical models of statistical analysis.

## Conclusions

The current study confirms that toxocariasis is a public health problem in urban areas of Fernandópolis, in northwestern São Paulo State, southeast Brazil. The presence of modifiable behaviors that increase soil ingestion, such as the habits of geophagy and a lack of hand washing, contributes to the seroprevalence rates observed in the evaluated schoolchildren.

## Supporting Information

Checklist S1 **STROBE Checklist**
(DOC)
