# Supplementary material for: Seroprevalence and Modifiable Risk Factors for Toxocara spp. in Brazilian Schoolchildren
Source: PLoS Negl Trop Dis. 2014 May 29;8(5):e2830. doi: 10.1371/journal.pntd.0002830 (PMC4038482; doi:10.1371/journal.pntd.0002830)
Supplement: Checklist S1 — STROBE Checklist (DOC) [file pntd.0002830.s001.doc]

**STROBE Statement—Checklist of items that should be included in reports of cross-sectional studies**

***Seroprevalence and modifiable risk factors for Toxocara spp. in Brazilian schoolchildren***

Alex J. F. Cassenote [1], Alba R. de Abreu Lima [2], José M. Pinto Neto [3], Guita Rubinsky-Elefant [4]

|  | Item No | Recommendation | Manuscript | Place in thus manuscript |
| --- | --- | --- | --- | --- |
| **Title and abstract** | 1 | (*a*) Indicate the study’s design with a commonly used term in the title or the abstract | **yes** | Page 2, line 1 |
|  |  | (*b*) Provide in the abstract an informative and balanced summary of what was done and what was found | **yes** | Page 2 line 3 to page 3 line 2 |
| Introduction | | |  |  |
| Background/rationale | 2 | Explain the scientific background and rationale for the investigation being reported | **yes** | Page 4 line 3 to page 5 line 14 |
| Objectives | 3 | State specific objectives, including any prespecified hypotheses | **yes** | Page 5 line 14 to 16 |
| Methods | | |  |  |
| Study design | 4 | Present key elements of study design early in the paper | **yes** | Page 5 line 22 |
| Setting | 5 | Describe the setting, locations, and relevant dates, including periods of recruitment, exposure, follow-up, and data collection | **yes** | Page 5 line 22 to page 6 line 8 |
| Participants | 6 | (*a*) Give the eligibility criteria, and the sources and methods of selection of participants | **yes** | Page 6 line 9 to page 7 line 10 |
| Variables | 7 | Clearly define all outcomes, exposures, predictors, potential confounders, and effect modifiers. Give diagnostic criteria, if applicable | **yes** | Page 7 line 6 to page 11 line 12 |
| Data sources/ measurement | 8* | For each variable of interest, give sources of data and details of methods of assessment (measurement). Describe comparability of assessment methods if there is more than one group | **yes** | Page 7 line 6 to page 11 line 12 |
| Bias | 9 | Describe any efforts to address potential sources of bias | **yes** | Page 7 line 6 to page 7 line 10 and  Page 11 line 21 to page 12 line 4 |
| Study size | 10 | Explain how the study size was arrived at | **yes** | Page 6 line 9 to 11 |
| Quantitative variables | 11 | Explain how quantitative variables were handled in the analyses. If applicable, describe which groupings were chosen and why | **Not applicable** | - |
| Statistical methods | 12 | (*a*) Describe all statistical methods, including those used to control for confounding | **yes** | Page 11 line 22 to page 12 line 15 |
|  |  | (*b*) Describe any methods used to examine subgroups and interactions | **yes** | Page 12, line 19 to 21 |
|  |  | (*c*) Explain how missing data were addressed | **Not applicable** | - |
|  |  | (*d*) If applicable, describe analytical methods taking account of sampling strategy | **yes** | Page 12 line 5 to page 12 line 10 |
|  |  | (*e*) Describe any sensitivity analyses | **no** | - |
| Results | | |  |  |
| Participants | 13* | (a) Report numbers of individuals at each stage of study—eg numbers potentially eligible, examined for eligibility, confirmed eligible, included in the study, completing follow-up, and analysed | **no** | - |
|  |  | (b) Give reasons for non-participation at each stage | **no** | - |
|  |  | (c) Consider use of a flow diagram | **no** | - |
| Descriptive data | 14* | (a) Give characteristics of study participants (eg demographic, clinical, social) and information on exposures and potential confounders | **yes** | Page 13 line 3 to page 14 line 10 |
|  |  | (b) Indicate number of participants with missing data for each variable of interest | **Not applicable** | - |
| Outcome data | 15* | Report numbers of outcome events or summary measures | **yes** | Page 13 line 3 to page 14 line 10 |
| Main results | 16 | (*a*) Give unadjusted estimates and, if applicable, confounder-adjusted estimates and their precision (eg, 95% confidence interval). Make clear which confounders were adjusted for and why they were included | **yes** | Page 13 line 21 to page 14 line 5 |
|  |  | (*b*) Report category boundaries when continuous variables were categorized | **Not applicable** | - |
|  |  | (*c*) If relevant, consider translating estimates of relative risk into absolute risk for a meaningful time period | **Not applicable** | - |
| Other analyses | 17 | Report other analyses done—eg analyses of subgroups and interactions, and sensitivity analyses | **Not applicable** | - |
| Discussion | | |  |  |
| Key results | 18 | Summarise key results with reference to study objectives | **yes** | Page 14 line 14 to page 18 line 23 |
| Limitations | 19 | Discuss limitations of the study, taking into account sources of potential bias or imprecision. Discuss both direction and magnitude of any potential bias | **yes** | Page 19 line 1 to page 19 line 20 |
| Interpretation | 20 | Give a cautious overall interpretation of results considering objectives, limitations, multiplicity of analyses, results from similar studies, and other relevant evidence | **yes** | Page 18 line 21 to page 19 line 11 |
| Generalisability | 21 | Discuss the generalisability (external validity) of the study results | **yes** | Page 14 line 21 to page 15 line 16 |
| Other information | | |  |  |
| Funding | 22 | Give the source of funding and the role of the funders for the present study and, if applicable, for the original study on which the present article is based | **Not applicable** | - |

*Give information separately for exposed and unexposed groups.

**Note:** An Explanation and Elaboration article discusses each checklist item and gives methodological background and published examples of transparent reporting. The STROBE checklist is best used in conjunction with this article (freely available on the Web sites of PLoS Medicine at http://www.plosmedicine.org/, Annals of Internal Medicine at http://www.annals.org/, and Epidemiology at http://www.epidem.com/). Information on the STROBE Initiative is available at www.strobe-statement.org.
